# Supplementary material for: Effectiveness of Roux-en-Y Gastric Bypass Versus Hypocaloric Diet in Reducing Cardiovascular Risk Factors in Obese Adults: A Systematic Review and Meta-Analysis
Source: J Clin Med. 2025 Nov 24;14(23):8349. doi: 10.3390/jcm14238349 (PMC12692916; doi:10.3390/jcm14238349)
Supplement: Supplementary file 1 [file jcm-14-08349-s001.zip › jcm-3972770-supplementary.pdf]

Comparison of Gastric Bypass vs. Hypocaloric Diet in Reducing Cardiovascular Risk Factors in Obese Adults: A Systematic Review with Meta-Analysis.

Autor: Darío S. López-Delgado[1]; Carlos A. Narvaez[2]; Nancy Doris Calzada Gonzales[3]; Rodrigo Martinez-Galaviz[4]; Oriana Rivera-Lozada[5], Joshuan J. Barboza[6].

SUPPLEMENTARY MATERIAL

Supplementary Figure S1. Risk of bias (RoB 2.0) for the randomized controlled trial 1
Supplementary Figure S2: Funnel plot 2
Supplementary Table S1: search strategies 3
Supplementary Table S2. Characteristics of included studies 5
Supplementary Table S3. Characteristics of interventions and control 8
Supplementary Table S4. Characteristics of included studies (data analysis) 10
Supplementary Table S5. Characteristics of included studies (statistical features) (1) 12
Supplementary Table S6. Characteristics of included studies (statistical features) (2) 13
Supplementary Table S7. Summary of Risk of Bias Assessment (NOS Scale) 14
Supplementary Table S8. GRADE Certainty of evidence 15
Supplementary Table S9: PRISMA 2020 Checklist 17

Supplementary Figure S1. Risk of bias (RoB 2.0) for the randomized controlled trial

Intention-to-treat	Unique ID	Study ID	Experimental	Comparator	Outcome	Weight	D1	D2	D3	D4	D5	Overall
	1_Study	Eriksson_2024	RYGB	low-calorie diet	LDL-cholesterol	1	+	+	+	+	+	+

+ Low risk
! Some concerns
- High risk

D1 Randomisation process
D2 Deviations from the intended interventions
D3 Missing outcome data
D4 Measurement of the outcome
D5 Selection of the reported result

Supplementary Figure S2: Funnel plot

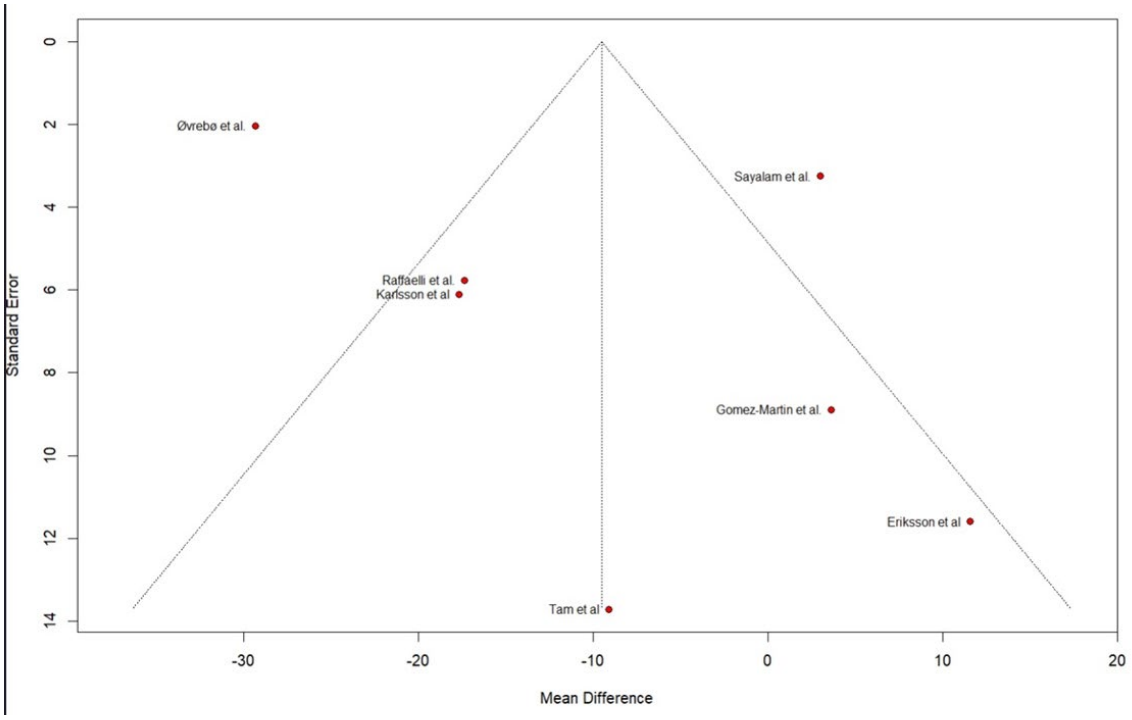

Supplementary Table S1: search strategies

**Database**	**PubMed**<br>**Search date:** May 28, 2025		**Results**
**Strategy**	#1	"Gastric Bypass"[Mesh] OR "Roux-en-Y Gastric Bypass" OR "RYGB" OR "Bariatric Surgery"[Mesh] OR "Gastrojejunostomy" OR "Gastroileal Bypass" OR "Surgical Weight Loss" OR "Bypass Surgery"	290
	#2	"hypocaloric diet" OR "caloric restriction" OR "low-calorie diet" OR "energy restriction" OR "dietary intervention" OR "diet modification" OR "diet therapy" OR "lifestyle intervention"	
	#3	"Cardiovascular Diseases"[Mesh] OR "Cardiovascular Risk Factors" OR "Cardiac Risk" OR "Atherosclerosis" OR "Lipid Profile" OR "Cholesterol" OR "LDL" OR "HDL" OR "Triglycerides" OR "Apolipoproteins" OR "lipoprotein" OR "Body Mass Index"[Mesh] OR "Blood Pressure"[Mesh] OR "Glycated Hemoglobin A"[Mesh]	
	#4	"Obesity"[Mesh] OR "Obese" OR "Overweight" OR "Adults with obesity"	
**Database**	**Web of Science**<br>**Search date:** May 28, 2025		**Results**
**Strategy**	#1	TS=("gastric bypass" OR "roux-en-y gastric bypass" OR "RYGB" OR "bariatric surgery" OR "gastrojejunostomy" OR "gastroileal bypass" OR "surgical weight loss" OR "bypass surgery")	505
	#2	TS=("hypocaloric diet" OR "caloric restriction" OR "low-calorie diet" OR "energy restriction" OR "dietary intervention" OR "diet modification" OR "diet therapy" OR "lifestyle intervention")	
	#3	TS=("cardiovascular disease" OR "cardiovascular risk factors" OR "cardiac risk" OR "atherosclerosis" OR "lipid profile" OR "cholesterol" OR "LDL" OR "HDL" OR "triglycerides" OR "apolipoproteins" OR "lipoprotein" OR "body mass index" OR "blood pressure" OR "HbA1c" OR "glycated hemoglobin")	
	#4	TS=("obesity" OR "obese" OR "overweight" OR "adults with obesity")	
**Database**	**Scopus**<br>**Search date:** May 28, 2025		**Results**
**Strategy**	#1	TITLE-ABS("gastric bypass" OR "roux-en-y gastric bypass" OR "RYGB" OR "bariatric surgery" OR "gastrojejunostomy" OR "gastroileal bypass" OR "surgical weight loss" OR "bypass surgery")	348
	#2	TITLE-ABS("hypocaloric diet" OR "caloric restriction" OR "low-calorie diet" OR "energy restriction" OR "dietary intervention" OR "diet modification" OR "diet therapy" OR "lifestyle intervention")	
	#3	TITLE-ABS("cardiovascular disease" OR "cardiovascular risk factors" OR "cardiac risk" OR "atherosclerosis" OR "lipid profile" OR "cholesterol" OR "LDL" OR "HDL" OR "triglycerides" OR "apolipoproteins" OR "lipoprotein" OR "body mass index" OR "blood pressure" OR "HbA1c" OR "glycated hemoglobin")	
	#4	TITLE-ABS("obesity" OR "obese" OR "overweight" OR "adults with obesity")	
**Database**	**EMBASE**<br>**Search date:** May 28, 2025		**Results**
**Strategy**	#1	'gastric bypass':ti,ab OR 'roux-en-y gastric bypass':ti,ab OR 'RYGB':ti,ab OR 'bariatric surgery':ti,ab OR 'gastrojejunostomy':ti,ab OR 'gastroileal bypass':ti,ab OR 'surgical weight loss':ti,ab OR 'bypass surgery':ti,ab	443
	#2	'hypocaloric diet':ti,ab OR 'caloric restriction':ti,ab OR 'low-calorie diet':ti,ab OR 'energy restriction':ti,ab OR 'dietary intervention':ti,ab OR 'diet modification':ti,ab OR 'diet therapy':ti,ab OR 'lifestyle intervention':ti,ab	
	#3	'cardiovascular disease':ti,ab OR 'cardiovascular risk factors':ti,ab OR 'cardiac risk':ti,ab OR 'atherosclerosis':ti,ab OR 'lipid profile':ti,ab OR 'cholesterol':ti,ab OR 'LDL':ti,ab OR 'HDL':ti,ab OR 'triglycerides':ti,ab OR 'apolipoproteins':ti,ab OR 'lipoprotein':ti,ab OR 'body mass index':ti,ab OR 'blood pressure':ti,ab OR 'HbA1c':ti,ab OR 'glycated hemoglobin':ti,ab	
	#4	'obesity':ti,ab OR 'obese':ti,ab OR 'overweight':ti,ab OR 'adults with obesity':ti,ab	

Supplementary Table S2. Characteristics of included studies

Author	Year	Country	Inclusion Criteria	Exclusion Criteria	number of participants	Age	Sex	Bypass Surgery Type	Complications
Gómez-Martin	2017	Spain	Severely obese women with high cardiovascular risk defined by presence of metabolic syndrome. BMI >45 for RYGB.	Mental impairment, uncontrolled psychiatric condition or substance abuse, active neoplastic disease, pregnancy, serious co-morbidities, treatment with thiazolidinediones.	40 (20 RYGB, 20 SG) + 20 controls (18 completed)	RYGB: 48±8, SG: 46±9, Controls: 52±7	Female	Laparoscopic Roux-en-Y gastric bypass (RYGB)	Not reported in detail; 1 RYGB patient had microvascular angina at 36 months
Øvrebø	2017	Norway	Age 18–65 years, BMI ≥40 or ≥35 kg/m² with comorbidity	Pregnancy, previous bariatric surgery, drug/alcohol abuse, severe psychiatric/physical disorders	209	Mean ~41 years	75% women	Roux-en-Y gastric bypass (RYGB)	Not specified
Raffaelli	2014	Italy	BMI ≥ 40 kg/m2 or ≥ 35 kg/m2 with T2DM; Age 30–60 years; no significant weight loss in past year	Previous major abdominal or bariatric surgery, cardiac or pulmonary diseases, cancer, corticosteroid use, mental illness	40	30–60 years	Surgical: 11W/9M; Diet: 12W/8M	Roux-en-Y gastric bypass (RYGB)	Not reported
Saiyalam	2024	Thailand	Age 15–65 years; BMI ≥ 37.5 kg/m² or ≥ 32.5 kg/m² with obesity-related complications (T2D, hypertension, dyslipidemia, sleep apnea, or NAFLD)	Type 1 diabetes, weight loss ≥5% in last 3 months, CKD (eGFR < 30), anti-obesity meds, pre/probiotics, uncontrolled psychiatric diseases, substance abuse, previous bariatric surgery, pregnancy/lactation, allergies to meal replacement	31		RYGB: 81.3% female, VLCD: 60% female	Laparoscopic Roux-en-Y Gastric Bypass	RYGB: abdominal discomfort (56.3%), nausea (43.8%), vomiting (25%), dumping (25%), constipation (25%); VLCD: fatigue (33.3%), sleepiness (33.3%), diarrhea (33.3%),

									nausea (13.3%), 1 excluded due to cholecystitis
Yoshino	2020	USA	Obese adults with type 2 diabetes; full criteria in Supplementary Appendix	Not detailed in main text; see Supplementary Appendix	22 analyzed (11 diet, 11 surgery); initially 33 enrolled		Diet group: 4 men, 7 women; Surgery group: 3 men, 8 women	Roux-en-Y gastric bypass (15-20 mL pouch, 75-150 cm Roux limb, 30-50 cm biliopancreatic limb)	Surgery group: 1 postoperative transfusion, 1 ED visit for food impaction; Diet group: none reported
Tam et al	2016	USA and Australia	People with obesity who self-selected for bariatric surgery or opted for a low-calorie diet; without diabetes diagnosed more than 5 years ago.	Potential participants who had diabetes diagnosed more than 5 years ago or had previous malabsorptive or restrictive surgery, a history of inflammatory intestinal disease, psychiatric conditions, or the use of medications that affect weight or metabolic rate were excluded.	Final data analyses were performed on 30 subjects (27 females; mean age, 46 2 years; mean body mass index, 47.2 1.5 kg/m2), of whom 21 subjects underwent scheduled bariatric surgery (five RYGB, nine SG, and seven LAGB) and nine subjects underwent a LCD intervention	mean age, 46 2 years	27 women and 3 men	Roux-en-Y gastric bypass (RYGB)	2 dropped out due to postoperative complications, but no details are provided.
Eriksson et al	2024	Suecia	Obese patients (BMI 35-45 kg/m²), without diabetes, on waiting list for bariatric surgery.	Exclusion criteria included diabetes, other endocrine disorders (except well-treated hypothyroidism), cancer, previous cardiovascular events, untreated sleep apnoea,	24 (15 Obesity Surgery, 9 LCD)	43 ± 11 years (OS), 41 ± 8 years (LCD)	1 male/14 female (OS), 1 male/8 female (LCD)	11 RYGB, 4 SG	None serious; 9 with symptoms of “dumping” after RYGB

				major illnesses and pregnancy.					
Karlsson et al	2024	Norway	Adults ≥18 years with severe obesity, stable body weight over the past 3 months, scheduled for RYGB or VLED.	Not detailed in the main text, but were evaluated by a physician according to pre-established criteria (see supplement).	78 (41 in RYGB, 37 in VLED)	RYGB group: 46.4 (9.4) VLED group (10.0)	65% women, 35% men	Laparoscopic Roux-en-Y gastric bypass (RYGB)	Not reported

Supplementary Table S3. Characteristics of interventions and control

Author	Year	Dietary Intervention in the control group (calories/day)	Dietary Intervention in the surgery group (calories/day)	Duration of Diet	Diet Adherence	Weight Change at 6 months	Weight Change at 12 months	Major Cardiovascular Events	Cardiovascular Mortality	Carotid Intima-media Thickness	Insulin Resistance
Gómez-Martin	2017	Diet and lifestyle modification; specific caloric intake not reported.	Not reported	1 year	Not specified	Not reported	EWL: RYGB: -72.4±13.3%, SG: -71.1±23.1%, Controls: -6.5±19.3%	1 control had a cerebrovascular event; 1 RYGB had microvascular angina	None reported	ΔIMT: RYGB: -0.08±0.09 mm, SG: -0.11±0.10 mm, Controls: +0.01±0.11 mm	ΔHOMA-IR: RYGB: -6.1±3.9, SG: -2.8±4.0, Controls: -0.03±2.05
Øvrebø	2017	Low-calorie diet at WL camp, 6 meals/day in residential program; specific caloric values not reported	Not specified	Varied: 16 weeks (WL camp), intermittent over 5 years (residential), 6+6 months (outpatient)	Not numerically reported	Not reported	RYGB: −30.4%, WL camp: −20.6%, Residential: −14.4%, Outpatient: −6.8%	Not reported	Not reported	Not reported	Glucose levels reported; RYGB improved more than lifestyle
Raffaelli	2014	15 kcal/kg/day (55% carbs, 30% fat, 15% protein) + 30 min brisk walk/day	Not reported	1 year	Not quantified, regular check-ins with diabetologist	Not numerically specified; weight stabilized at 9 months post-RYGB	RYGB: -37 kg; Diet: -8 kg	Not measured (surrogate markers only)	Not measured		HOMA-IR and OGIS improved significantly in RYGB group
Saiyalam	2024	VLCD: 800–900 kcal/day, 96–120 g protein	RYGB: 300–700 kcal/day, 20–44 g protein	12 weeks	High during 12 weeks; follow-up VLCD: 60% at 12 months	VLCD: some regain; RYGB: all maintained	VLCD: some regain; 3/15 needed intervention; RYGB: maintained	Not reported	Not reported	Not reported	FPG and HbA1c improved in both groups

Yoshino	2020	Liquid shakes and prepackaged entrees; exact kcal not detailed	Adjusted post-surgery to meet weight loss goals; exact kcal not detailed	Until 16–24% weight loss achieved (~2091±136 kcal/day during maintenance)	High; weight maintained within 1.2% variability before final assessments	Not specified by timepoint; mean loss: diet group 17.8±1.2%, surgery group 18.7±2.5%	Not applicable; study endpoint was matched weight loss (~18%)	Not reported	Not reported	Not reported	Improvements in hepatic, muscle, and adipose insulin sensitivity; no significant group differences
Tam et al	2016	800 kcal/day for 8 weeks, then a maintenance diet with -500 kcal according to energy expenditure.	890 kcal/day during week 8 assessment	8 initial intensive weeks, then maintenance diet for 1 year (LCD group)	Not specified in detail, but patients in the diet group attended weekly and then monthly visits.	Not reported	RYGB: −33% LCD: −4%	Not reported	Not reported	Not reported	HOMA-IR significantly improved in RYGB (-3.2), LCD group not reported.
Eriksson et al	2024	1 100 kcal/day liquids during 4 weeks	None prescribed prior to surgery	4 weeks	15/15 OS and 9/9 LCD	Not reported	Not reported	Not reported	Not reported	Not reported	HOMA-IR was significantly reduced following OS (−26.3% [95% CI −49.5, −3.0], p=0.009); the reduction was not statistically significant following LCD (−20.9% [95% CI −58.2, 16.5]).
Karlsson et al	2024	<800 kcal/day for 6 weeks	Same, <800 kcal/day for 6 weeks after RYGB.	3 weeks LED + 6 weeks VLED	Monitored with 4-day dietary diary; good adherence in both groups.	Not reported	Not reported	Not reported	Not reported	Not reported	Assessed by HOMA2%S, no differences between groups.

Supplementary Table S4. Characteristics of included studies (data analysis)

Author	Year	Confounding Variables	Type of Analysis	Outcomes
Gómez-Martin	2017	Baseline differences in BMI, insulin resistance, statin use	Repeated measures GLM, ANOVA, χ^2 test	Both RYGB and SG significantly reduced carotid IMT and improved metabolic/cardiovascular risk markers compared to controls.
Øvrebø	2017	Baseline differences in sex, weight, BMI; self-reported comorbidities; no randomization	Observational study, intention-to-treat, completers analysis	RYGB led to greater long-term weight loss, improved glucose and HDL, and hypertension remission compared to lifestyle
Raffaelli	2014	Statin use, smoking, diabetes prevalence	Wilcoxon, Mann-Whitney U, Spearman correlation, multiple regression	HDL-C and ApoA4 increased significantly post-RYGB; improved insulin sensitivity; ApoA4 changes associated with weight loss
Saiyalam	2024	Non-randomized design; patient selection based on preference; T2D duration, motivation, health literacy	Multilevel mixed-effects linear regression; t-test; quantile regression; Fisher's exact test	Similar weight loss, %WL, T2D remission (RYGB: 57%, VLCD: 80%); VLCD preserved muscle mass better; RYGB had better long-term weight maintenance
Yoshino	2020	Non-randomized design; participant self-selection; dropout differences	ANCOVA with baseline and group assignment as predictors; hyperinsulinemic euglycemic clamp; mixed-meal tests	Weight loss improved beta-cell function, insulin sensitivity, body composition; similar improvements between diet and surgery; distinct metabolite and microbiome changes post-surgery not associated with superior outcomes
Tam et al	2016	No randomization, small but not significant baseline differences; self-selected surgery (possible selection bias).	Prospective observational study, parallel group analysis.	Glucose, Insulin, HOMA-IRscore, Triglycerides, Totalcholesterol, HDL, LDL, HDL-to-LDLratio, flammation Totaladiponectin, HMWadiponectin, Leptin, T3, T4, TSH
Eriksson et al	2024	No masking, OS/LCD ratio changed from 1:1 to 2:1, small sample size	Parallel randomized clinical trial, ANCOVA analysis adjusted for baseline.	Body Mass Index, body weight, body weight loss (%), waist circumference, hip circumference, waist/hip ratio, lean body mass (LBM) %, body fat %, whole-body volume, adipose tissue volume, non-adipose tissue volume, liver fat %, pancreas fat %, heart rate, systolic blood pressure, diastolic blood pressure, HbA1c (glycated hemoglobin), fasting plasma glucose, HOMA-IR (Homeostatic Model Assessment for Insulin Resistance), fasting S-insulin, clamp insulin, 2h post-OGTT glucose, insulinogenic index, disposition index, Matsuda index, M value (whole-body insulin sensitivity during clamp), plasma creatinine, plasma

Karlsson et al	2024	Adjustments for age, sex, BMI and baseline variable in mixed models	Non-randomized, controlled, mixed-model analysis for repeated measures	ALT (alanine aminotransferase), plasma AST (aspartate aminotransferase), plasma ALP (alkaline phosphatase), plasma C-reactive protein, plasma cholesterol, plasma HDL-cholesterol, plasma LDL-cholesterol, plasma triglycerides, total physical activity (light, moderate, vigorous), energy expenditure (24 hours), GIR (glucose infusion rate), EGP (endogenous glucose production), Rd (whole-body glucose disposal), MRglu brain (glucose uptake rate in brain), MRglu liver (glucose uptake rate in liver), MRglu heart (glucose uptake rate in heart), MRglu abdominal adipose tissue (glucose uptake rate in abdominal adipose tissue), MRglu leg muscles (glucose uptake rate in skeletal muscles of legs).<br>Body Mass Index, Body Weight, Fat-Free Mass, Fat Mass, Waist Circumference, Waist-Hip Ratio, Glycated Hemoglobin (HbA1c), Fasting Glucose, Fasting Insulin, C-peptide, HOMA2%S (Homeostasis Model Assessment estimate of insulin sensitivity), HOMA2%B (Homeostasis Model Assessment estimate of beta-cell function), hepatic insulin clearance, total cholesterol, LDL cholesterol (low-density lipoprotein cholesterol), HDL cholesterol (high-density lipoprotein cholesterol), triglycerides, non-HDL cholesterol, apolipoprotein B, lipoprotein(a), LDL cholesterol/apolipoprotein B ratio, systolic blood pressure, diastolic blood pressure, pulse/heart rate, hs–C-reactive protein (high-sensitivity C-reactive protein), leptin, FGF19 (Fibroblast Growth Factor 19), creatinine, hemoglobin.

Supplementary Table S5. Characteristics of included studies (statistical features) (1)

		LDL-C											HDL-C											BODY MASS INDEX						
**Author**	**Year**	Definition	Ne	Me	Se	Nc	Mc	Sc	lie	Ise	lic	Isc	Definition	Ne	Me	Se	Nc	Mc	Sc	lie	Ise	lic	Isc	Definition	Ne	Me	Se	Nc	Mc	Sc
Gomez-Martin et al. (1 years)	2017	mg/dL	20	–1.6	30.6	18	–5.2	24.1					mg/dL	20	8.5	9.8	18	0.3	5.8					kg/m2	20	–12.4	3.9	18	–1.3	4.2
Øvrebø et al. (1 years)	2017	mmol/l	58	2.25	0.14	30	03.01	0.27	1.99	2.52	2.49	3.54	mmol/l	58	1.41	0.05	58	1.61	0.10	1.31	1.51	1.41	1.81							
Øvrebø et al. (5 years)	2017	mmol/l	58	2.54	0.13	30	3.28	0.28	2.29	2.79	2.73	3.82	mmol/l	30	1.64	0.05	30	1.33	0.11	1.55	1.74	1.12	1.54							
Raffaelli et al. (1 years)	2014	mg/dL	20	-19.3	16.78	20	-1.94	19.54					mg/dL	20	14.6	5.98	20	2.81	4.34					kg/m2	20	-12.06	3.61	20	-2	4.31
Saiyalam et al. (4 weeks)	2024																							kg/m2	16	-3.3	7.78	15	-2.1	6.78
Saiyalam et al. (12 weeks)	2024	mg/dL	16	−25.0	6.38	15	−28.0	10.97	−34.0	−9.0	−35.0	8.0	mg/dL	16	−3.0	4.46	15	−5.0	3.57	10.0	7.5	−13.0	1.0	kg/m2	16	-6.7	7.78	15	-5.5	6.78
Yoshino et al. (3 weeks)	2020																							kg/m2	11	-8.1	2.51	11	-7.7	4.58
Tam et al (1 year)	2016	mg/dL	5	-0.3	21.01	9	8.8	30	0.07	-0.67	-14.26	31.86	mg/dL	5	12.2	14.98	9	-0.7	6.3	-2.95	27.35	-5.54	4.14							
Tam et al (8 weeks)	2016	mg/dL	5	3.6	27.06	9	-3.9	20.7	-0.87	8.06	-19.81	12.01	mg/dL	5	-9.6	12.97	9	-5.1	6.6	2.31	-21.52	-10.17	-0.03							
Eriksson et al (4 Weeks)	2024	mmol/l	15	-0.7	0.7	9	-1	0.72	-0.31	-1.08	-1.55	-0.44	mmol/l	15	-0.25	0.29	9	-0.17	0.23	-0.11	-0.38	-0.34	0.0037	kg/m2	15	-3.1	2.4	9	-2.9	2.75
Karlsson et al (3 Weeks)	2024	mg/dL	41	-23.36	18.33	37	-18	18.03	-28.97	-17.75	-23.81	-12.19	mg/dL	41	-6.554	5.68	37	-6.602	5.61	-8.292	-4.82	-8.41	-4.794	kg/m2	41	-2.07	0.95	37	-2.05	0.93
Karlsson et al (9 Weeks)	2024	mg/dL	41	-13.31	23.06	37	4.37	29.98	-20.37	-6.25	-2.98	11.71	mg/dL	41	0.422	6.08	37	1.121	6.02	-1.44	2.283	-0.818	3.06	kg/m2	41	-3.62	0.83	37	-2.77	0.81

Supplementary Table S6. Characteristics of included studies (statistical features) (2)

Author	Year	SYSTOLIC BLOOD PRESSURE							TRIGLYCERIDES											HbA1c										
		Definition	Ne	Me	Se	Nc	Mc	Sc	Definition	Ne	Me	Se	Nc	Mc	Sc	lie	Ise	lic	Isc	Definition	Ne	Me	Se	Nc	Mc	Sc	lie	Ise	lic	Isc
Gomez-Martin et al. (1 years)	2017	mmHg	20	–12.1	23.0*	18	–6.6	18.8	mg/dL	20	–61.8	88.5	18	–16.7	58.1															
Øvrebø et al. (1 years)	2017								mmol/l	58	0.98	0.12	30	01.01	0.23	0.75	1.21	0.58	1.50											
Øvrebø et al. (5 years)	2017								mmol/l	58	1.17	0.11	30	1.32	0.24	0.95	1.38	0.84	1.80											
Raffaelli et al. (1 years)	2014	mmHg	20	-15.2	10.10	20	-18.4	16.07	mg/dL	20	-75.45	59.81	20	-15.92	58.19					HbA1c, %	20	-1.89	1.54	20	-0.12	0.69				
Saiyalam et al. (4 weeks)	2024																													
Saiyalam et al. (12 weeks)	2024								mg/dL	16	-38.0	20.79	15	-22.0	13.01	-69.5	12.0	-54.0	-3.0	HbA1c, %	16	-0.5	0.15	15	-0.4	0.26	−0.9	−0.3	-1.0	0.0
Yoshino et al. (3 weeks)	2020																			HbA1c, %	11	-1.2	0.90	11	-2.4	1.64				
Tam et al (1 year)	2016								mg/dL	5	-60.4	89.89	9	6.1	17.1	14.6	-135.39	-7.04	19.24											
Tam et al (8 weeks)	2016								mg/dL	5	-29	80.05	9	-18.7	47.4	7.01	-65.01	-55.13	13.73											
Eriksson et al (4 Weeks)	2024	mmHg	15	-10	13.45	9	-12	14.11	mmol/l	15	-0.16	0.29	9	-0.44	0.57	-0.32	0	-0.88	0	HbA1c, %	15	-0.38	0.28	9	-0.17	0.4	-0.17	-0.6	-0.48	-0.14
Karlsson et al (3 Weeks)	2024	mmHg	41	-8.5	9.64	37	-6.8	9.47	mg/dL	41	-50.62	47.39	37	-51.77	47.16	-65.13	-36.12	-66.96	-36.57	HbA1c, %	41	-0.25	0.25	37	-0.31	0.29	-0.32	-0.17	-0.39	-0.23
Karlsson et al (9 Weeks)	2024	mmHg	41	-1.9	5.63	37	2.6	4.76	mg/dL	41	-4.12	19.46	37	8.75	81.03	-22.18	13.93	-17.36	34.86	HbA1c, %	41	-0.37	0.29	37	-0.32	0.29	-0.46	-0.28	-0.42	-0.23

Supplementary Table S7. Summary of Risk of Bias Assessment (NOS Scale)

Study	Selection	Comparability	Outcome	Conclusion
Gómez-Martin (2017)	★★★★	★★	★★★	Low risk
Yoshino (2020)	★★★	★★	★★★	Low risk
Tam (2016)	★★★★	★★	★★★	Low risk
Øvrebø (2017)	★★★★	★★	★★★	Low risk
Karlsson (2024)	★★★★	★★	★★★	Low risk
Raffaelli (2014)	★★★★	★★	★★★	Low risk
Saiyalam (2024)	★★★★	★★	★★★	Low risk

Supplementary Table S8. GRADE Certainty of evidence

Roux-en-Y Gastric Bypass compared to Hypocaloric Diet in Reducing Cardiovascular Risk Factors in Obese Adults

Bibliography:

**Certainty assessment**							**Summary of findings**				
							**Study event rates (%)**			**Anticipated absolute effects**	
**Participants (studies) Follow-up**	**Risk of bias**	**Inconsistency**	**Indirectness**	**Imprecision**	**Publication bias**	**Overall certainty of evidence**	**With Hypocaloric Diet**	**With Roux-en-Y Gastric Bypass**	**Relative effect (95% CI)**	**Risk with Hypocaloric Diet**	**Risk difference with Roux-en-Y Gastric Bypass**
**LDL cholesterol (follow-up: mean 12 months; assessed with: mg/dL)**											
313 (7 non-randomised studies)	not serious	serious[a]	not serious	serious[b]	none	⊕◯◯◯ Very low[a,b]	138	175	-	138	MD **24.13 mg/dL lower** (37.02 lower to 11.24 lower)
**HDL cholesterol (follow-up: mean 12 months; assessed with: mg/dL)**											
341 (7 non-randomised studies)	not serious	serious[c]	not serious	serious[d]	none	⊕◯◯◯ Very low[c,d]	166	175	-	166	MD **2.81 mg/dL higher** (2.83 lower to 8.45 higher)
**Triglycerides (follow-up: mean 12 months; assessed with: mg/dL)**											
313 (7 non-randomised studies)	not serious	serious[e]	not serious	serious[f]	none	⊕◯◯◯ Very low[e,f]	138	175	-	138	MD **18.58 mg/dL lower** (37.71 lower to 0.54 higher)
**Body Mass Index (follow-up: mean 12 months; assessed with: kg/m²)**											

Roux-en-Y Gastric Bypass compared to Hypocaloric Diet in Reducing Cardiovascular Risk Factors in Obese Adults

Bibliography:

Certainty assessment							Summary of findings				
233 (6 non-randomised studies)	not serious	serious[g]	not serious	serious[h]	none	⊕◯◯◯ Very low[g,h]	110	123	-	110	MD **4.02 kg/m^2 lower** (8.16 lower to 0.12 higher)
**Hemoglobin A1c (follow-up: mean 12 months; assessed with: %)**											
195 (5 non-randomised studies)	not serious	serious[i]	not serious	serious[j]	none	⊕◯◯◯ Very low[i,j]	92	103	-	92	MD **0.22 % lower** (1.09 lower to 0.65 higher)
**Systolic blood pressure (follow-up: mean 12 months; assessed with: mmHg)**											
180 (4 non-randomised studies)	not serious	not serious	not serious	serious[k]	none	⊕◯◯◯ Very low[k]	84	96	-	84	MD **2.77 mm Hg lower** (6.39 lower to 0.85 higher)

CI: confidence interval; **MD:** mean difference

Supplementary Table S9: PRISMA 2020 Checklist.

Section and Topic	Item #	Checklist item	Location where item is reported/Page #
**TITLE**			
Title	1	Identify the report as a systematic review.	1
**ABSTRACT**			
Abstract	2	See the PRISMA 2020 for Abstracts checklist.	1
**INTRODUCTION**			
Rationale	3	Describe the rationale for the review in the context of existing knowledge.	1-2
Objectives	4	Provide an explicit statement of the objective(s) or question(s) the review addresses.	1-2
**METHODS**			
Eligibility criteria	5	Specify the inclusion and exclusion criteria for the review and how studies were grouped for the syntheses.	3
Information sources	6	Specify all databases, registers, websites, organisations, reference lists and other sources searched or consulted to identify studies. Specify the date when each source was last searched or consulted.	3
Search strategy	7	Present the full search strategies for all databases, registers and websites, including any filters and limits used.	3
Selection process	8	Specify the methods used to decide whether a study met the inclusion criteria of the review, including how many reviewers screened each record and each report retrieved, whether they worked independently, and if applicable, details of automation tools used in the process.	3
Data collection process	9	Specify the methods used to collect data from reports, including how many reviewers collected data from each report, whether they worked independently, any processes for obtaining or confirming data from study investigators, and if applicable, details of automation tools used in the process.	3
Data items	10a	List and define all outcomes for which data were sought. Specify whether all results that were compatible with each outcome domain in each study were sought (e.g. for all measures, time points, analyses), and if not, the methods used to decide which results to collect.	3
	10b	List and define all other variables for which data were sought (e.g. participant and intervention characteristics, funding sources). Describe any assumptions made about any missing or unclear information.	3
Study risk of bias assessment	11	Specify the methods used to assess risk of bias in the included studies, including details of the tool(s) used, how many reviewers assessed each study and whether they worked independently, and if applicable, details of automation tools used in the process.	3
Effect measures	12	Specify for each outcome the effect measure(s) (e.g. risk ratio, mean difference) used in the synthesis or presentation of results.	3

Section and Topic	Item #	Checklist item	Location where item is reported/Page #
Synthesis methods	13a	Describe the processes used to decide which studies were eligible for each synthesis (e.g. tabulating the study intervention characteristics and comparing against the planned groups for each synthesis (item #5)).	3
	13b	Describe any methods required to prepare the data for presentation or synthesis, such as handling of missing summary statistics, or data conversions.	3
	13c	Describe any methods used to tabulate or visually display results of individual studies and syntheses.	3
	13d	Describe any methods used to synthesize results and provide a rationale for the choice(s). If meta-analysis was performed, describe the model(s), method(s) to identify the presence and extent of statistical heterogeneity, and software package(s) used.	3
	13e	Describe any methods used to explore possible causes of heterogeneity among study results (e.g. subgroup analysis, meta-regression).	3
	13f	Describe any sensitivity analyses conducted to assess robustness of the synthesized results.	3
Reporting bias assessment	14	Describe any methods used to assess risk of bias due to missing results in a synthesis (arising from reporting biases).	3
Certainty assessment	15	Describe any methods used to assess certainty (or confidence) in the body of evidence for an outcome.	4
**RESULTS**			
Study selection	16a	Describe the results of the search and selection process, from the number of records identified in the search to the number of studies included in the review, ideally using a flow diagram.	4
	16b	Cite studies that might appear to meet the inclusion criteria, but which were excluded, and explain why they were excluded.	4
Study characteristics	17	Cite each included study and present its characteristics.	4-6
Risk of bias in studies	18	Present assessments of risk of bias for each included study.	9
Results of individual studies	19	For all outcomes, present, for each study: (a) summary statistics for each group (where appropriate) and (b) an effect estimate and its precision (e.g. confidence/credible interval), ideally using structured tables or plots.	6
Results of syntheses	20a	For each synthesis, briefly summarise the characteristics and risk of bias among contributing studies.	6-9
	20b	Present results of all statistical syntheses conducted. If meta-analysis was done, present for each the summary estimate and its precision (e.g. confidence/credible interval) and measures of statistical heterogeneity. If comparing groups, describe the direction of the effect.	6-9
	20c	Present results of all investigations of possible causes of heterogeneity among study results.	6-9
	20d	Present results of all sensitivity analyses conducted to assess the robustness of the synthesized results.	6-9

Section and Topic	Item #	Checklist item	Location where item is reported/Page #
Reporting biases	21	Present assessments of risk of bias due to missing results (arising from reporting biases) for each synthesis assessed.	9
Certainty of evidence	22	Present assessments of certainty (or confidence) in the body of evidence for each outcome assessed.	9-10
**DISCUSSION**			
Discussion	23a	Provide a general interpretation of the results in the context of other evidence.	10
	23b	Discuss any limitations of the evidence included in the review.	11-12
	23c	Discuss any limitations of the review processes used.	12
	23d	Discuss implications of the results for practice, policy, and future research.	12
**OTHER INFORMATION**			
Registration and protocol	24a	Provide registration information for the review, including register name and registration number, or state that the review was not registered.	2
	24b	Indicate where the review protocol can be accessed, or state that a protocol was not prepared.	2
	24c	Describe and explain any amendments to information provided at registration or in the protocol.	-
Support	25	Describe sources of financial or non-financial support for the review, and the role of the funders or sponsors in the review.	12
Competing interests	26	Declare any competing interests of review authors.	13
Availability of data, code and other materials	27	Report which of the following are publicly available and where they can be found: template data collection forms; data extracted from included studies; data used for all analyses; analytic code; any other materials used in the review.	12
